# Differentiating between severe hypertension and thrombocytopenic purpura as the cause of thrombotic microangiopathy in a patient with Castleman disease: a case report and literature review

**DOI:** 10.1093/omcr/omad059

**Published:** 2023-06-26

**Authors:** Itamar Feldman, Dvorah S Shapiro, Raphael Ellis, Reuven Friedman

**Affiliations:** Intermediate Care Department, Bait Balev Hospital, Rishon LeZion, Israel; Department of Geriatrics, Shaare Zedek Medical Center, Jerusalem, Israel; Division of Internal Medicine, Shaare Zedek Medical Center, Jerusalem, Israel; Hadassah Hebrew University School of Medicine, Jerusalem Israel; Department of Geriatrics, Shaare Zedek Medical Center, Jerusalem, Israel; Division of Internal Medicine, Shaare Zedek Medical Center, Jerusalem, Israel; Department of Geriatrics, Shaare Zedek Medical Center, Jerusalem, Israel; Division of Internal Medicine, Shaare Zedek Medical Center, Jerusalem, Israel; Hadassah Hebrew University School of Medicine, Jerusalem Israel

## Abstract

Thrombotic microangiopathy (TMA) is a syndrome characterized by thrombosis in capillaries and arterioles, resulting in microangiopathic hemolytic anemia, thrombocytopenia and target organ injury. In TMA presenting with severe hypertension, it is difficult to determine whether the TMA is primary and due to thrombotic thrombocytopenic purpura (TTP) or secondary to severe hypertension. The response to antihypertensive medication favors the diagnosis of severe hypertension as the cause of TMA. Comorbid inflammatory disease supports the diagnosis of TTP-induced TMA. This case describes a 75-year-old woman with Castleman disease who presented with severe hypertension and TMA. She improved with hypertension therapy. However, ADAMST13 showed zero activity, and the diagnosis was TTP. In cases of TMA accompanied by severe hypertension, it is challenging to diagnose the cause of TMA. Even when there is a pronounced clinical response to lowering blood pressure, the diagnosis of TTP should be considered, particularly when an inflammatory disease is present.

## INTRODUCTION

Thrombotic microangiopathy (TMA) is a syndrome characterized by thrombosis in capillaries and arterioles together with a clinical presentation of microangiopathic hemolytic anemia (MAHA), thrombocytopenia and target organ injury. There are many causes of TMA, but the main categories are divided into primary and secondary. Primary TMA includes thrombotic thrombocytopenic purpura (TTP), hemolytic uremic syndrome, complement-mediated TMA and cobalamin deficiency. Each of these types has a specific deficiency or impairment that should be identified and corrected to resolve the TMA [1, 2]. Secondary TMA is a process secondary to underlying systemic disease. Examples of secondary TMA causes are severe hypertension, infection, malignancy and autoimmune diseases. TMA is treated by diagnosing and treating the underlying disorder. Recognizing TMA as a medical emergency, prompt identification of its specific subtype and initiation of appropriate treatment are critical to minimize organ damage and enhance survival [3, 4].

Differentiating between TTP and severe hypertension as the cause of TMA in patients who present with hypertension, thrombocytopenia, MAHA and target organ injury is difficult. In this paper, we present such a case, review the literature for similar cases and discuss the appropriate way to manage similar patients.

### Case presentation

**Figure 1:**
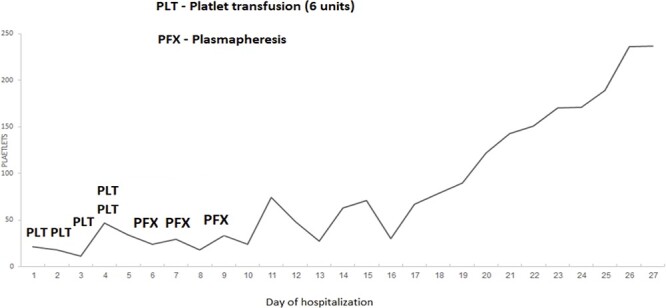
Change in PLT count during hospitalization relative to PLT transfusions and plasmapheresis treatment.

A 75-year-old woman was admitted to the emergency department with abdominal pain and headache. Two years prior to her admission, she was diagnosed with multicentric Castleman disease (MCD), which had remained stable. It was characterized by lymphadenopathy and mild thrombocytopenia, and no treatment had been administered during this time. In addition, her medical history included hypertension, atrial fibrillation, congestive heart failure, chronic renal failure, ischemic heart disease and cerebrovascular disease. Upon admission, her blood pressure (BP) was 240/111 mmHg; all other vital signs were normal (N). Her blood test revealed chronic microcytic anemia (hemoglobin, 9; MCV, 66), acute thrombocytopenia [platelets (PLTs), 21 000/μl], elevated bilirubin (2.7, UNL < 1.5 mg/dl), elevated lactate dehydrogenase (600 IU/L, UNL < 220) and undetectable haptoglobin. White blood cells, creatinine and coagulation parameters were within normal range, and Coombs test was negative. An ADAMST13 activity blood test was drawn and sent to the lab. Subsequently, neurologic signs developed: right hand weakness, full blindness, with a consciousness level rapidly deteriorating to a comatose state. Fundus examination showed exudative retinal detachment without tear. Numerous schistocytes were evident on a peripheral blood film. In suspicion of posterior reversible encephalopathy syndrome, computed tomography and magnetic resonance imaging (MRI) tests of the brain were conducted and did not demonstrate acute findings. The combination of thrombocytopenia, MAHA, the mentioned retinal findings and neurological signs, along with excessive hypertension, led to a diagnosis of hypertensive emergency with secondary TMA. Intravenous labetalol treatment was initiated, and her BP was gradually stabilized. After several hours, she became fully alert and all neurological signs resolved but the thrombocytopenia worsened (PLT 11 000/μl). Two days later, the result of the ADAMST13 blood test returned showing zero activity. Subsequently, plasmapheresis treatment was initiated (3 treatments) with a gradual improvement of PLT count to 236 000/μl (Fig. 1). The patient was discharged with steroid treatment and hematology follow-up. Two months later, she presented with mild thrombocytopenia (PLT 90 000/μl), and schistocytes were observed in the blood film. Rituximab treatment was added and the PLT count gradually rose to within normal range.

## DISCUSSION

TMA is a medical emergency and should be rapidly diagnosed and treated. However, diagnosing the cause of TMA can be challenging, even for experienced clinicians. A study of 254 physicians found that 47% diagnose the underlying condition of TMA after >1 week of presentation, and 13% initiate treatment beyond 1 week post presentation [5].

TTP is the most common cause of primary TMA. Impaired activity of ADAMST13, a von Willebrand factor-cleaving protease, causes thrombi formation in small vessels and the clinical presentation of TMA. The most common underlying etiology is IgG antibodies formed against ADAMST13 [5].

Without plasmapheresis treatment, TTP has the highest mortality rate of all etiologies of TMA (90%) [6]; therefore, rapid diagnosis and treatment initiation are necessary. The diagnosis of TTP is confirmed by measurement of ADAMST13 activity. However, this test is not available in every hospital, and there can be a delay of days until the clinician receives the results. Therefore, in cases highly suspicious for TTP, as well as in cases of TMA without other identifiable etiology, plasmapheresis should be initiated, even without a definitive diagnosis [1, 2, 6].

Hypertensive emergency is an umbrella term for clinical conditions characterized by excessive hypertension presenting with target organ damage [7]. One of these conditions is hypertensive encephalopathy, a syndrome of extreme hypertension and neurological deficits such as altered mental status, visual deficits and seizures. Hypertensive encephalopathy is characterized by cerebral edema and increased intracranial pressure following the failure of autoregulation mechanisms of brain vessels against excessively high arterial pressure [8]. The hallmark of hypertensive encephalopathy is the resolution of clinical symptoms and radiological signs following BP decrease [9].

As both hypertension-induced TMA and TTP may have similar clinical presentations, differentiating between the two can be complex. The case presented here concerns a female patient with a background of MCD who presented with a clinical and laboratory picture of TMA. Given that her presentation included severe hypertension with notable neurological signs that dramatically resolved upon BP stabilization, TMA secondary to severe hypertension was the suspected diagnosis; however, the final diagnosis was TTP.

Several case reports and discussions have addressed the challenge of differentiating between hypertension and TTP-induced TMA in patients who present with excessive hypertension and clinical presentation of TMA. The consensus is that patients with TMA should be managed with empiric plasmapheresis (PFX). Nevertheless, when there is a highly suggestive precipitating factor for TMA, and there is dramatic clinical improvement upon treatment, it is reasonable to delay PFX.

Khanal *et al*. reviewed the literature for cases of hypertension-induced TMA and found that they are characterized by high mean atrial pressure (160 mmHg), median PLT count of 59 000/μl and significant acute kidney injury (AKI; average creatinine, 4.14 mg/dl). The most prominent finding was that all patients showed symptomatic improvement after lowering their BP [10]. Abdalla *et al*. described a 63-year-old patient who presented with dyspnea, hematemesis, epistaxis, blurred vision, severe hypertension (200/106 mmHg) and unremarkable findings in fundus exam. Laboratory results revealed hemolytic anemia, thrombocytopenia (PLT 40 000/μl) and markedly elevated creatinine (13.5 mg/dl). Numerous schistocytes per HPF were observed in peripheral blood smear, and the ADAMTS13 activity test showed 69% activity. PFX was started immediately, and there was profound AKI that required hemodialysis. The ADAMST13 test result did not support TTP, and the final diagnosis was malignant hypertension [11]. Mitaka *et al*. describe a 49-year-old male with a history of hypertension who presented with excessive hypertension (240/140 mmHg), AKI (CRT 11 mg/dl), thrombocytopenia (97 000/μl) and schistocytes in blood smear. Fundoscopy revealed hypertensive retinopathy, and MRI demonstrated posterior reversible encephalopathy. As the findings were highly suggestive of hypertension-induced TMA, the patient was treated with antihypertensive agents and hemodialysis, with subsequent dramatic improvement in his PLT counts and improved kidney function. ADAMST13 activity was 61%, excluding the diagnosis of TTP [12]. Shibagaki *et al*. described a 33-year-old male who presented with severe hypertension (160/120 mmHg) after discontinuing his antihypertensive medication. He was found to have thrombocytopenia (69 000/μl), MAHA and severe AKI requiring hemodialysis. Fundus examination revealed significant cotton exudates in both retinas. He was treated with intravenous antihypertensive agents with clinical and laboratory improvement. PFX was not initiated, and ADAMST13 activity was normal. The authors suggested that the immediate decision should rely on PLT counts. When there is severe thrombocytopenia (PLT < 5000/μl), empiric PFX should be imitated. Otherwise, the decision should be based on the clinical situation, including the degree of hypertension and history of longstanding hypertension, which favor the diagnosis of hypertension-induced TMA [13]. Based on one case, Zheng *et al*. suggested using immature PLT increments following PFX initiation as a support for the diagnosis of TTP-induced TMA. However, this method requires initiation of PFX prior to the final diagnosis [14].

As the most common cause of TTP is antibodies against ADAMST13, a presentation of TMA in patients with a background of autoimmune or inflammatory diseases may favor the diagnosis of TTP-induced TMA. Given that MCD is characterized by overproduction of cytokines and immunoglobulins [15], it is reasonable to assume that MCD may be associated with TTP. Indeed, there are anecdotal reports of an association between CD and TTP. Benevides  *et al*. described the first case of ADAMST13 deficiency and TTP precipitated by unicentric Castleman disease [16]. London *et al*. described four cases of TTP associated with MCD [17].

In summary, we described a case of a patient with MCD who presented with TMA. The initial presentation was highly suggestive of TMA secondary to severe hypertension. Despite the dramatic clinical response to hypertension treatment, ADAMST13 activity was null and the final diagnosis was TTP.

## CONCLUSION

Identifying the specific subtype of TMA rapidly and initiating the correct treatment is essential. In cases of TMA accompanied by severe hypertension, it is challenging to correctly diagnose the cause of TMA. Regardless of a dramatic clinical response to lowering BP, the diagnosis of TTP should still be considered, especially when there is a comorbid inflammatory disease.

## CONFLICT OF INTEREST STATEMENT

None declared.

## ETHICAL APPROVAL

Ethical approval was not required for this study.

## CONSENT

The patient provided written consent upon admission, acknowledging that their case may be used for academic purposes, including publication.

## GUARANTOR

Itamar Feldman is the guarantor of this article.
